# Supplementary figures and images for: Capture-based enrichment of Theileria parva DNA enables full genome assembly of first buffalo-derived strain and reveals exceptional intra-specific genetic diversity
Source: PLoS Negl Trop Dis. 2020 Oct 29;14(10):e0008781. doi: 10.1371/journal.pntd.0008781 (PMC7654785; doi:10.1371/journal.pntd.0008781)

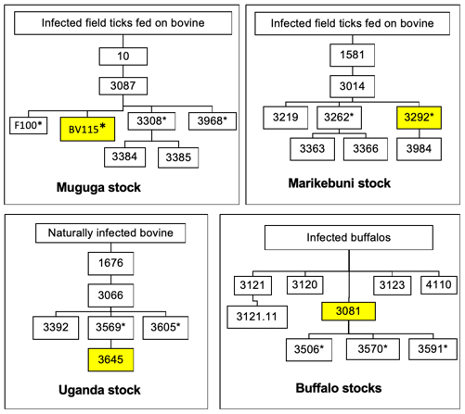

Supplement: S1 Fig — Stabilate numbers are shown. An * indicates stabilates generated using a 3-step cloning procedure as described by Morzaria and colleagues [64]. Stabilates used in this study are colored in yellow. The last step of the preparation of stabilates (except for 3121.11) included tick pick-up on infected cow or buffalo [64]. (PNG) [file pntd.0008781.s001.png]

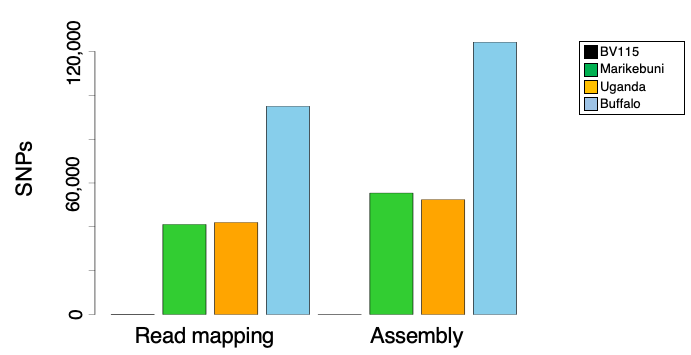

Supplement: S2 Fig — The total number of SNPs identified was compared between read-mapping and assembly approaches, for each of the four isolates. As expected, almost no SNPs were found between BV115 (animal infected with the Muguga strain) and the reference T. parva Muguga genome assembly. Identification of SNPs based on assembly alignment is consistently more sensitive than red mapping. Twice as many SNPs are found in the buffalo- than in cattle- derived strain relative to the Muguga reference, from cattle. (PNG) [file pntd.0008781.s002.png]

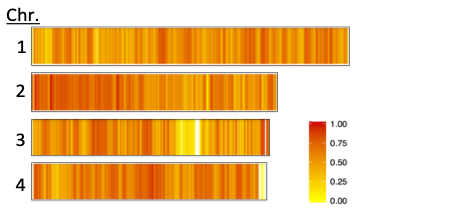

Supplement: S3 Fig — Window-based FST analysis comparing cattle strains to those derived from buffalo. Analysis includes our strains and those from Hayashida et al. (2013). The windows used were 4,000 bp long with a 1,000 bp overlapping window. Genome-wide FST was 0.436. (PNG) [file pntd.0008781.s003.png]

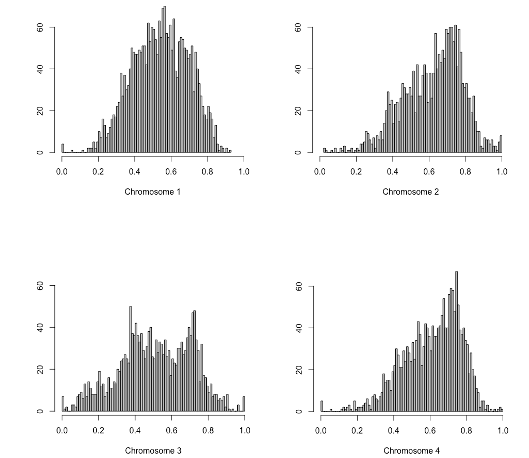

Supplement: S4 Fig — Histograms of window-based FST values calculated for each nuclear chromosome, showing a wide range of FST values throughout the genome. Frequency of average FST value is shown on x-axis, FST values shown on y-axis. (PNG) [file pntd.0008781.s004.png]
