# Supplementary material for: Capture-based enrichment of Theileria parva DNA enables full genome assembly of first buffalo-derived strain and reveals exceptional intra-specific genetic diversity
Source: PLoS Negl Trop Dis. 2020 Oct 29;14(10):e0008781. doi: 10.1371/journal.pntd.0008781 (PMC7654785; doi:10.1371/journal.pntd.0008781)
Supplement: S1 Table — Properties of gaps in probe coverage across all assembly contigs and supercontigs (chromosomes) and across protein-coding genes. (DOCX) [file pntd.0008781.s005.docx]

**Supplemental Table S1. Probe Coverage Statistics.** Properties of gaps in probe coverage across all assembly contigs and supercontigs (chromosomes) and across protein-coding genes.

|  |  |  | Genomic Regions Not Mapped by Probes | | | | | | | Gene Mapping by Probes | | | |
| --- | --- | --- | --- | --- | --- | --- | --- | --- | --- | --- | --- | --- | --- |
| Chromosome | Contig | Contig Length (bp) | Number of gaps | Median length (bp) | Mean length (bp) | StdDev (bp) | Min length (bp) | Max length (bp) | Cumulative (bp) | Total Genes (#) | Genes  Not Covered (#) | Partially Covered Genes (#) | Fully Covered Genes (#) |
| 1 | AAGK01000001 | 2,540,030 | 1,183 | 17 | 63 | 325 | 2 | 7,131 | 74,943 | 1224 | 4 | 570 | 650 |
| 2 | AAGK01000002 | 1,971,884 | 935 | 19 | 81 | 257 | 2 | 4,448 | 76,092 | 984 | 7 | 462 | 515 |
| 3 | AAGK01000005 | 1,317,241 | 755 | 21 | 74 | 206 | 2 | 3,945 | 56,002 | 635 | 0 | 313 | 322 |
| 3 | AAGK01000006 | 570,487 | 329 | 23 | 200 | 866 | 2 | 7,210 | 65,647 | 283 | 14 | 139 | 130 |
| 3 | AAGK01000007 | 41,585 | 22 | 126 | 1,569 | 2,559 | 7 | 8,690 | 34,507 | 18 | 9 | 9 | 0 |
| 3 | AAGK01000008 | 13,275 | 1 | 13,275 | 13,275 | - | 13,275 | 13,275 | 13,275 | 6 | 6 | 0 | 0 |
| 4 | AAGK01000004 | 1,835,834 | 762 | 14 | 46 | 130 | 2 | 2,010 | 35,032 | 934 | 3 | 397 | 534 |
| 4 | AAGK01000003 | 17,691 | 21 | 153 | 670 | 1,083 | 16 | 3,808 | 14,069 | 10 | 4 | 6 | 0 |
| Apicoplast | AAGK01000009 | 39,579 | 29 | 8 | 204 | 490 | 2 | 1,860 | 5,910 | 70 | 6 | 22 | 42 |
| Total | 9 | 8,347,606 | 4,037 | 6 | 87 | 454 | 2 | 52,377 | 375,477 | 4,164 | 53 | 1,918 | 2,193 |
