# Supplementary material for: Capture-based enrichment of Theileria parva DNA enables full genome assembly of first buffalo-derived strain and reveals exceptional intra-specific genetic diversity
Source: PLoS Negl Trop Dis. 2020 Oct 29;14(10):e0008781. doi: 10.1371/journal.pntd.0008781 (PMC7654785; doi:10.1371/journal.pntd.0008781)
Supplement: S2 Table — Properties related to library construction and generation of whole genome sequence data. (DOCX) [file pntd.0008781.s006.docx]

**Supplemental Table S2. Sample metadata.** Properties related to library construction and generation of whole genome sequence data.

| **Isolate** | **Sequencing Platform** | **Starting Material (ηg)** | **Average gDNA shearing size (bp)** | **Average Size of Captured Fragment** | **Mean Read Length (bp)** | **Reads generated** |
| --- | --- | --- | --- | --- | --- | --- |
| BV115 | Illumina HiSeq 2000 | 900 | 500 | 446 | 101 | 12,174,316 |
| Marikebuni | Illumina MiSeq | 900 | 600-700 | 533 | 250 | 7,204,556 |
| Uganda | Illumina MiSeq | 900 | 600-700 | 555 | 247 | 5,687,838 |
| Buffalo_3081 | Illumina MiSeq | 1,200 | 600-700 | 619 | 249 | 6,080,972 |
