# Supplementary material for: Capture-based enrichment of Theileria parva DNA enables full genome assembly of first buffalo-derived strain and reveals exceptional intra-specific genetic diversity
Source: PLoS Negl Trop Dis. 2020 Oct 29;14(10):e0008781. doi: 10.1371/journal.pntd.0008781 (PMC7654785; doi:10.1371/journal.pntd.0008781)
Supplement: S3 Table — (DOCX) [file pntd.0008781.s007.docx]

**Supplemental Table S3. Sequence variants identified by read mapping relative to the reference *T. parva* Muguga genome assembly.**

|  | ***T. parva* Muguga genome with coverage that satisfies SNP calling filter** | **Sequence variants *vs*. reference *T. parva* Muguga genome^1^** | |
| --- | --- | --- | --- |
| **Isolate** | **Base pairs (%)** | **SNPs** | **INDELs** |
| BV115 | 99.50 | 107 | 18 |
| Marikebuni | 96.81 | 40,228 | 11 |
| Uganda | 97.48 | 40,835 | 7 |
| Buffalo_3081 | 95.61 | 91,840 | 4 |

^1^Sequence variants, including single nucleotide polymorphisms (SNPs) and small insertions and deletions (INDELs) were identified using the Genome Analysis Toolkit.
