# Supplementary material for: Capture-based enrichment of Theileria parva DNA enables full genome assembly of first buffalo-derived strain and reveals exceptional intra-specific genetic diversity
Source: PLoS Negl Trop Dis. 2020 Oct 29;14(10):e0008781. doi: 10.1371/journal.pntd.0008781 (PMC7654785; doi:10.1371/journal.pntd.0008781)
Supplement: S4 Table — (DOCX) [file pntd.0008781.s008.docx]

**Supplemental Table S4. Assembly validation and correction.**

| **Validation Test** | **BV115** | **Marikebuni** | **Uganda** | **Buffalo _3081** |
| --- | --- | --- | --- | --- |
| Length of orthologous reference assembly^1^ | 8,347,606 | 8,635,931 | 8,388,202 | NA |
| # base pairs in orthologous reference genome^1^ with 0X coverage by capture reads |  | 119,137 | 60,471 | NA |
| Capture sensitivity relative to orthologous reference genome^1^ |  | 98.6% | 99.3% | NA |
|  |  |  |  |  |
| # SNPs when aligning reads to respective *de novo* assembly | 73 | 320 | 183 | 431 |
| # SNPs in *de* novo assembly corrected with Pilon | 50 | 139 | 78 | 117 |
| # SNPs when aligning reads to respective orthologous reference assembly^1^ | 107 | 203 | 79 | NA |
| # SNPs when aligning *de novo* assemblies against reference assembly^1^ | 92 | 96 | 165 | NA |
| # SNPs when aligning *de novo*, Pilon-corrected assemblies against orthologous reference assembly^1^ | 61 | 85 | 158 | NA |

^1^ References used: for BV115 the reference Muguga; for Marikebuni and Uganda, the respective 454-based assemblies in Henson *et al*. (2012). NA: not applicable; no orthologous reference is available for *T. parva* Buffalo_3081, so these statistics could not be obtained.
