# Supplementary material for: Capture-based enrichment of Theileria parva DNA enables full genome assembly of first buffalo-derived strain and reveals exceptional intra-specific genetic diversity
Source: PLoS Negl Trop Dis. 2020 Oct 29;14(10):e0008781. doi: 10.1371/journal.pntd.0008781 (PMC7654785; doi:10.1371/journal.pntd.0008781)
Supplement: S5 Table — Assembly length, number of contigs and gene content are shown. Gene content was assessed with two approaches: “read mapping” and “assembly alignment”. (DOCX) [file pntd.0008781.s009.docx]

**Supplemental Table S5. Gene content in *de novo* *T. parva* genome assemblies.** Assembly length, number of contigs and gene content are shown. Gene content was assessed with two approaches: “read mapping”^1^ and “assembly alignment”^2^.

| **Sample** | **Assembly Length (bp)** | **# Scaffolds** | **Complete Nuclear Genes** | | **Partial Nuclear Genes** | | **Absent Nuclear Genes** | | ***T. parva* Reference Genome Aligned (%)** |
| --- | --- | --- | --- | --- | --- | --- | --- | --- | --- |
|  |  |  | **Assembly Alignment** | **Read Mapping** | **Assembly Alignment** | **Read Mapping** | **Assembly Alignment** | **Read Mapping** |  |
| BV115 | 8,236,259 | 123 | 4,015 | 4,094 | 72 | 0 | 7 | 0 | 98.05 |
| Marikebuni | 8,274,714 | 116 | 4,024 | 3,917 | 84 | 147 | 56 | 30 | 96.18 |
| Uganda | 8,277,618 | 126 | 4,020 | 3,922 | 89 | 128 | 31 | 44 | 96.16 |
| Buffalo_3081 | 8,366,826 | 109 | 4,017 | 3,871 | 96 | 198 | 27 | 25 | 95.68 |

^1^Proportion of each gene in the reference genome assembly with read coverage from each isolate. ^2^Proportion of each gene in the reference genome that is aligned with an orthologous section in the de novo assembly of each non-reference isolate. These results were split into three categories: genes with complete coverage, genes partially covered, and genes that are completely absent from the assembly.
