# Supplementary material for: Capture-based enrichment of Theileria parva DNA enables full genome assembly of first buffalo-derived strain and reveals exceptional intra-specific genetic diversity
Source: PLoS Negl Trop Dis. 2020 Oct 29;14(10):e0008781. doi: 10.1371/journal.pntd.0008781 (PMC7654785; doi:10.1371/journal.pntd.0008781)
Supplement: S6 Table — (DOCX) [file pntd.0008781.s010.docx]

**Supplemental Table S6. Homology searches for predicted *Theileria parva* genes in unmapped contigs.**

|  | **Strain** | | | |
| --- | --- | --- | --- | --- |
|  | **BV115** | **Marikebuni** | **Uganda** | **Buffalo_3081** |
| Total Unmapped contigs | 93 | 61 | 72 | 82 |
| Contigs with no defined orthology to the reference *T. parva* genome | 6 | 9 | 7 | 12 |
| **Gene family** |  | | | |
| SVSP family protein | 39 | 24 | 28 | 17 |
| Tpr family protein | 28 | 28 | 0 | 30 |
| TpHN family protein | 14 | 7 | 0 | 9 |
| DEAD/DEAH box helicase | 0 | 0 | 1 | 2 |
| hypothetical protein | 24 | 41 | 64 | 67 |
| ABC transporter | 7 | 12 | 4 | 12 |
| Other | 16 | 15 | 34 | 28 |
| **Total** | 128 | 127 | 131 | 165 |

*Portions of the above gene families were found in the unmapped contigs
