# Supplementary material for: Capture-based enrichment of Theileria parva DNA enables full genome assembly of first buffalo-derived strain and reveals exceptional intra-specific genetic diversity
Source: PLoS Negl Trop Dis. 2020 Oct 29;14(10):e0008781. doi: 10.1371/journal.pntd.0008781 (PMC7654785; doi:10.1371/journal.pntd.0008781)
Supplement: S7 Table — (DOCX) [file pntd.0008781.s011.docx]

**Supplemental Table S7. Structural variants in *de novo* assemblies compared to reference assemblies^1^.**

|  | **BV115** | | **Marikebuni** | | **Uganda** | |
| --- | --- | --- | --- | --- | --- | --- |
| **Variant type** | **Count** | **Total bp** | **Count** | **Total bp** | **Count** | **Total bp** |
| **Insertion** | 2 | 171 | 0 | 0 | 0 | 0 |
| **Deletion** | 1 | 128 | 1 | 51 | 0 | 0 |
| **Tandem expansion** | 10 | 5,206 | 9 | 914 | 5 | 373 |
| **Tandem contraction** | 6 | 1,099 | 1 | 87 | 0 | 0 |
| **Repeat expansion** | 1 | 69 | 0 | 0 | 0 | 0 |
| **Repeat contraction** | 6 | 4,715 | 0 | 0 | 0 | 0 |
| **Total for all variants** | 26 | 11,388 | 11 | 1,052 | 5 | 373 |

**^1^** BV115 assembly was compared to the reference *T. parva* Muguga genome (Gardner et al. 2005). The assemblies for Marikebuni and Uganda were aligned to respective references previously generated with 454 data (Henson et al. 2012). Insertions and deletions correspond to interruptions in regions that are otherwise syntenic. Tandem expansions and contractions occur between overlapping variants, whereas repeat expansions and contractions occur within unmappable gaps between alignments.
