# Supplementary material for: Capture-based enrichment of Theileria parva DNA enables full genome assembly of first buffalo-derived strain and reveals exceptional intra-specific genetic diversity
Source: PLoS Negl Trop Dis. 2020 Oct 29;14(10):e0008781. doi: 10.1371/journal.pntd.0008781 (PMC7654785; doi:10.1371/journal.pntd.0008781)
Supplement: S8 Table — (DOCX) [file pntd.0008781.s012.docx]

**Supplemental Table S8. Structural variants between *T. parva* strains^1^.**

|  | **Marikebuni** | | **Uganda** | | **Buffalo_3081** | |
| --- | --- | --- | --- | --- | --- | --- |
| **Variant type** | **Count** | **Total bp** | **Count** | **Total bp** | **Count** | **Total bp** |
| **Insertion** | 32 | 2,955 | 26 | 3,363 | 83 | 17,534 |
| **Deletion** | 19 | 1,725 | 25 | 2,332 | 66 | 18,941 |
| **Tandem expansion** | 10 | 10,840 | 13 | 16,340 | 26 | 20,000 |
| **Tandem contraction** | 6 | 6,794 | 6 | 8,819 | 8 | 1,142 |
| **Repeat expansion** | 64 | 25,151 | 67 | 23,080 | 71 | 24,098 |
| **Repeat contraction** | 69 | 31,027 | 60 | 24,394 | 78 | 47,035 |
| **Total for all variants** | 200 | 78,492 | 197 | 78,328 | 333 | 128,750 |

**^1^** Each assembly was compared to the reference *T. parva* Muguga genome (Gardner et al. 2005).
