# Supplementary material for: Capture-based enrichment of Theileria parva DNA enables full genome assembly of first buffalo-derived strain and reveals exceptional intra-specific genetic diversity
Source: PLoS Negl Trop Dis. 2020 Oct 29;14(10):e0008781. doi: 10.1371/journal.pntd.0008781 (PMC7654785; doi:10.1371/journal.pntd.0008781)
Supplement: S9 Table — (DOCX) [file pntd.0008781.s013.docx]

**Supplemental Table S9. Best BLAST match for each *T. parva* Buffalo_3081 gene without a detectable homolog in *T. parva* Muguga.**

| **Species** | **Accession** | **Product name** | **e-value** | **length** |
| --- | --- | --- | --- | --- |
| *Theileria annulata* | XM_946869.1 | mitochondrial ribosomal protein S14 precursor | 5.89E-122 | 448 |
| *Theileria annulata* | XM_948711.1 | hypothetical protein | 8.90E-159 | 571 |
| *Theileria annulata* | XM_949468.1 | hypothetical protein | 1.53E-82 | 316 |
| *Theileria annulata* | XM_947853.1 | tRNA-pseudouridine synthase I | 1.39E-168 | 604 |
| *Theileria annulata* | XM_947854.1 | hypothetical protein | 0 | 776 |
| *Theileria orientalis* | AP011947.1 | Match to chromosome 2 | 8.74E-31 | 145 |
| *Theileria parva* | XM_758627.1 | hypothetical protein | 6.54E-139 | 505 |
| *Theileria parva* | XM_758396.1 | hypothetical protein | 0 | 2,122 |
| *Theileria parva* | XM_758138.1 | hypothetical protein | 1.7E-37 | 167 |
| *Theileria parva* | XM_758263.1 | Major Facilitator Superfamily protein | 0 | 911 |
| *Theileria parva* | XM_758222.1 | hypothetical protein | 4.72E-50 | 211 |
| *Theileria parva* | XM_758205.1 | Papain family cysteine protease family protein | 0 | 1,373 |
| *Theileria parva* | XM_758149.1 | putative integral membrane protein | 6.89E-111 | 411 |
| *Theileria parva* | XM_761650.1 | hypothetical protein | 0 | 1421 |
| *Theileria parva* | XM_757752.1 | hypothetical protein | 1.37E-129 | 473 |
| *Theileria parva* | XM_759519.1 | hypothetical protein | 0 | 1,225 |
| *Theileria parva* | XM_760276.1 | hypothetical protein | 0 | 774 |
| *Theileria parva* | XM_761390.1 | EAP30/Vps36 fmaily protein | 0 | 1,092 |
| *Theileria parva* | XM_760316.1 | putative integral membrane protein | 0 | 704 |
| *Theileria parva* | XM_759574.1 | Papain family cysteine protease family protein | 0 | 1,358 |
| *Theileria parva* | XM_761509.1 | haloacid dehalogenase-like hydrolase family protein | 0 | 983 |
| *Theileria parva* | XM_761113.1 | hypothetical protein | 0 | 1,301 |
| *Theileria parva* | XM_760000.1 | hypothetical protein | 2.18E-78 | 305 |
| *Theileria parva* | XM_758820.1 | Translation initiation factor IF-2 | 1.4E-26 | 132 |
| *Theileria parva* | XM_759449.1 | hypothetical protein | 0 | 1,105 |
