# Supplementary material for: Capture-based enrichment of Theileria parva DNA enables full genome assembly of first buffalo-derived strain and reveals exceptional intra-specific genetic diversity
Source: PLoS Negl Trop Dis. 2020 Oct 29;14(10):e0008781. doi: 10.1371/journal.pntd.0008781 (PMC7654785; doi:10.1371/journal.pntd.0008781)
Supplement: S11 Table — (DOCX) [file pntd.0008781.s015.docx]

**Supplemental Table S11. Summary statistics of nucleotide diversity among cattle-derived *T. parva* strains, and between cattle and the buffalo-derived strain *T. parva* Buffalo_3081 shown in Supplemental Table 10.**
