# Supplementary material for: Capture-based enrichment of Theileria parva DNA enables full genome assembly of first buffalo-derived strain and reveals exceptional intra-specific genetic diversity
Source: PLoS Negl Trop Dis. 2020 Oct 29;14(10):e0008781. doi: 10.1371/journal.pntd.0008781 (PMC7654785; doi:10.1371/journal.pntd.0008781)
Supplement: S13 Table — (DOCX) [file pntd.0008781.s017.docx]

**Supplemental Table S13. Detection of rapidly evolving genes^1^.**

| **Product name** | **Locus tag**  **(for individual genes)** | Top *π***_N_** among cattle | Top *π***_N_** between Muguga and Buffalo_3081 | Top *π*_N_/*π*_S_ between Muguga and Buffalo_3081 |
| --- | --- | --- | --- | --- |
| 5'-3' exoribonuclease 1 | TpMuguga_02g00165 |  | X |  |
| ABC transporter | TpMuguga_01g00646 TpMuguga_02g00016 TpMuguga_02g00951 TpMuguga_03g00007  TpMuguga_03g00864 | X  X  X  X  X |  |  |
| Adaptin N terminal region | TpMuguga_04g00106 | X |  |  |
| Ankyrin repeat family protein | TpMuguga_03g00538 | X |  |  |
| AP2 domain protein | TpMuguga_03g00093 |  |  | X |
| Archease protein family (MTH1598/TM1083) | TpMuguga_03g00010 |  | X | X |
| Beta-Casp domain | TpMuguga_03g00560 | X |  |  |
| Biotin-requiring enzyme family protein | TpMuguga_03g00320 |  | X | X |
| Box C/D snoRNA protein 1 | TpMuguga_03g02410 |  |  | X |
| Chaperone protein DnaJ | TpMuguga_01g02410 |  | X |  |
| Choline/ethanolamine kinase | TpMuguga_02g00655 | X |  |  |
| Chromosome segregation protein Spc25 family protein | TpMuguga_03g00851 |  |  | X |
| Chymosin | TpMuguga_03g02555 |  | X |  |
| Cyclin-dependent kinase regulatory subunit family protein | TpMuguga_03g00089 |  | X | X |
| Cytokine-induced anti-apoptosis inhibitor 1 Fe-S biogenesis | TpMuguga_01g00461 |  | X | X |
| DnaJ domain protein | TpMuguga_02g00414 |  | X |  |
| DSHCT (NUC185) domain protein | TpMuguga_04g00364 |  | X |  |
| ELM2 domain protein | TpMuguga_01g00312 |  | X |  |
| EMG1/NEP1 methyltransferase | TpMuguga_03g00619 | X | X |  |
| Epsin-2 | TpMuguga_01g00558 |  | X |  |
| eRF1 methyltransferase catalytic subunit MTQ2 | TpMuguga_04g00566 | X |  |  |
| Eukaryotic glutathione synthase ATP binding domain | TpMuguga_01g00265  TpMuguga_01g00264 | X  X |  |  |
| Exonuclease 1 | TpMuguga_04g00145 | X | X |  |
| Exosome complex component RRP45 | TpMuguga_01g00013 |  |  | X |
| GDP dissociation inhibitor family protein | TpMuguga_04g00080 |  | X |  |
| Haemolysin-III related family protein | TpMuguga_04g00201 |  |  | X |
| Haloacid dehalogenase-like hydrolase  **Haloacid dehalogenase-like hydrolase**  **Haloacid dehalogenase-like hydrolase** | TpMuguga_01g01075  **TpMuguga_01g01078**  **TpMuguga_01g01081** | **X** | X | **X** |
| Histone H3-like centromeric protein CSE4 | TpMuguga_02g00044 |  |  | X |
| Hypothetical protein | **TpMuguga_03g00263** | 31A  40AB  5AC  16ABC | 21B  40AB  8BC  16ABC | 68C  5AC  8BC  16ABC  **X** |
| Leucine carboxyl methyltransferase family protein | TpMuguga_02g00808 | X | X |  |
| Metallopeptidase family M24 | TpMuguga_03g00462 |  |  | X |
| Mitochondrial large subunit ribosomal protein (Img2) family protein | TpMuguga_03g02335 | X | X |  |
| Myb-like DNA-binding domain protein | TpMuguga_02g00403 |  | X | X |
| NADPH:adrenodoxin oxidoreductase mitochondrial | TpMuguga_02g02525 |  | X |  |
| Nucleoplasmin family protein | TpMuguga_04g00909 |  | X |  |
| **p104 - 104 kDa microneme/rhoptry antigen** | **TpMuguga_04g00437** |  |  | **X** |
| **p32 - Merozoite Antigen** | **TpMuguga_01g01056** | **X** |  | **X** |
| **PIM** | **TpMuguga_04g00051** | **X** | **X** | **X** |
| Polyubiquitin | TpMuguga_02g00142 |  |  | X |
| Pre-mRNA splicing factor family protein | TpMuguga_01g01190 | X | X | X |
| Pre-mRNA-splicing factor of RES complex | TpMuguga_02g00881 |  | X |  |
| Pre-rRNA-processing protein esf-2 | TpMuguga_01g00309 |  | X |  |
| Protein kinase domain protein | TpMuguga_02g00630 |  |  | X |
| Putative integral membrane protein |  | 9A  7AB  1AC  4ABC | 8B  7AB  3BC  4ABC | 13C  3BC  1AC  4ABC |
| Rab-GTPase-TBC domain protein | TpMuguga_04g00105 | X | X | X |
| Reactive mitochondrial oxygen species modulator 1 family protein | TpMuguga_04g00826 | X |  |  |
| Replication factor RFC1 C terminal domain | TpMuguga_03g00565 | X | X |  |
| Ribosomal protein L7/L12 C-terminal domain protein | TpMuguga_03g00328 |  |  | X |
| RNA polymerase II subunit A C-terminal domain phosphatase | TpMuguga_03g00826 |  | X |  |
| RNAse P Rpr2/Rpp21/SNM1 subunit domain protein | TpMuguga_01g00916 |  |  | X |
| S-adenosyl-L-methionine-dependent tRNA 4-demethylwyosine synthase | TpMuguga_01g00125 |  | X |  |
| Sas10/Utp3/C1D family protein | TpMuguga_01g02625 |  |  | X |
| SEP domain protein | TpMuguga_03g00300 | X |  |  |
| Sin3 associated polypeptide p18 (SAP18) family protein | TpMuguga_03g00824 |  | X | X |
| SVSP family protein | **TpMuguga_01g01225**  **TpMuguga_02g00958** | 9A  6AB  3AC  2ABC | 4B  6AB  10BC  2ABC | 16C  3AC  10BC  2ABC  **X**  **X** |
| **Tash protein PEST motif family protein** | **TpMuguga_04g00164** | X | X | X |
| Telomere recombination | TpMuguga_03g00474 | X |  |  |
| **Tp1** | **TpMuguga_03g00849** |  |  | **X** |
| **Tp2** | **TpMuguga_01g00056** | **X** | **X** | **X** |
| **Tp9** | **TpMuguga_02g00895** | **X** | **X** | **X** |
| TpHN family protein | TpMuguga_01g00609  TpMuguga_01g00615  TpMuguga_01g00616  TpMuguga_01g00605  TpMuguga_01g00610  TpMuguga_01g00607  TpMuguga_01g00619  TpMuguga_01g00602 | X |  | X  X  X  X  X  X  X |
| Tpr family protein |  | 3A  28AB  1ABC | 1B  28AB  1BC  1ABC | 0C  1BC  1ABC |
| Trafficking protein particle complex subunit 10, TRAPPC10 family protein | TpMuguga_02g00868 | X | X |  |
| Transcription factor/nuclear export subunit protein 2 family protein | TpMuguga_02g00884 |  | X | X |
| Translation initiation factor 1A / IF-1 family protein | TpMuguga_01g00597 |  |  | X |
| Translation initiation factor IF-2 | TpMuguga_01g01188  TpMuguga_01g02835  TpMuguga_04g00278  TpMuguga_04g00279  TpMuguga_04g00280 |  | X  X | X  X  X  X |
| Type-2 histone deacetylase 2 | TpMuguga_03g02515 | X | X |  |
| U3 small nucleolar RNA-associated protein 6 family protein | TpMuguga_03g00023 | X |  |  |
| Ubiquitin family protein | TpMuguga_01g00315 | X |  |  |
| Urm1 (Ubiquitin related modifier) family protein | TpMuguga_02g00852 |  | X | X |
| Utp11 protein | TpMuguga_01g00183 |  |  | X |
| WD domain, G-beta repeat family protein | TpMuguga_01g00545 | X |  |  |
| Ydr279p protein family (RNase H2 complex component) | TpMuguga_03g00595 |  |  | X |
| Zinc finger A20 and AN1 domain-containing stress-associated protein 9  Zinc finger A20 and AN1 domain-containing stress-associated protein 9 | TpMuguga_04g00113  TpMuguga_04g00117 |  |  | X  X |
| Zn-finger in Ran binding protein and others family protein | TpMuguga_04g00822 | X | X |  |

^1^Genes encoding the proteins with the highest rate of amino acid polymorphism (top 200 *π***_N_** values) among cattle strains (Muguga, Marikebuni and Uganda) and between cattle (Muguga) and buffalo (Buffalo_3081) strains, and the 200 genes with the highest *π*_N_/*π*_S_, ratio between cattle (Muguga) and buffalo (LAWR) strains. Rows with an “X” indicate an individual gene within one or multiple classes, where “A”, “B”, and “C” are used when more than eight genes have the same product name. “A” indicates genes falling in the Top *π***_N_** among cattle class, “B” indicates Top *π***_N_** between Muguga and Buffalo_3081, and “C” indicates Top *π*_N_/*π*_S_ between Muguga and Buffalo_3081. If a gene falls in multiple classes, the respective letters are used.
