# Supplementary material for: Capture-based enrichment of Theileria parva DNA enables full genome assembly of first buffalo-derived strain and reveals exceptional intra-specific genetic diversity
Source: PLoS Negl Trop Dis. 2020 Oct 29;14(10):e0008781. doi: 10.1371/journal.pntd.0008781 (PMC7654785; doi:10.1371/journal.pntd.0008781)
Supplement: Attachment — Submitted filename: Manuscript.docx [file pntd.0008781.s018.docx]

Manuscript : PNTD-D-20-01054

Title : Capture-based enrichment of *Theileria parva* DNA enables full genome assembly of first buffalo-derived strain and reveals exceptional intra-specific genetic diversity

Authors : Palmateer *et al.*

The authors report the first use of capture-based target enrichment to sequence and assemble the genomes of Thieleria parva parasites from infected mammalian cells. Although this approach has been previously applied to sequencing the Plasmodium genome (e.g. Bright et al. 2012), several features of the experimental design for Theileria have resulted in impressive levels of specificity and sensitivity and coverage.

The sequencing data allowed de novo genome assembly providing an important proof-of-concept and a resource for future studies of parasite genotypes and population genetics. The generation of the first buffalo-derived T.parva genome is interesting because of the distinct infection symptoms and could be relevant to vaccine development. The sequencing data reveal differences in the genome size and genetic diversity between cattle- and buffalo-derived parasites.

Minor points

- It is a shame that the authors only tested their approach on established infected cell lines. They should at least comment on the feasibility of testing on clinical samples from infected cows and ticks with respect to sensitivity and the limited sample material. They should also comment on the cost, time-requirement and scalability of this technology.
- Figure 2 is so small that the images will be hardly readable in the final print form.
- Page 19: the authors report 24 new genes found in the Buffalo assembly. Are the 19 genes, representing copies of T. parva genes, found in particular locations adjacent to their orthologues? Are the 5 genes with T. annulata and T.orientalis homologues founds in regions syntenic with the other species? All these 24 genes should be listed in a table with T.annulata and T.orientalis reference names.
- Page 28: The manuscript should have consistent citation (by numbers) e.g. lines 685, 686, 687 etc.
- The authors should comment on the occurrence and predicted effects of specific SNPs in parasite genes encoding proteins shown to be important for host-parasite interactions and drug targeting: e.g. p67, Tp1, Tp2, UB05, Pin1, cytochrome B, p104, Ta9, MISHIP, TashAT2 etc.
