# Supplementary material for: Capture-based enrichment of Theileria parva DNA enables full genome assembly of first buffalo-derived strain and reveals exceptional intra-specific genetic diversity
Source: PLoS Negl Trop Dis. 2020 Oct 29;14(10):e0008781. doi: 10.1371/journal.pntd.0008781 (PMC7654785; doi:10.1371/journal.pntd.0008781)
Supplement: Attachment — Submitted filename: Palmateer-etal_2020-08-24_reviewer_comments.docx [file pntd.0008781.s019.docx]

**We are very thankful to all three reviewers for their careful and useful comments which, we believe, resulted in an improved manuscript.**

Editor's comments:

   line 165; can you not tell from the SNPs in the Muguga_3087 and Buffalo_7014 genome sequences whether they are clonal or not?

**Reply: This clonality description refers to the initial processing of the isolates upon collection, prior to this study, as described in Morzaria et al*.* (1995). Even though Muguga_3087 was not clonal, BV115 (sequenced here) is. We now include a more comprehensive description of the history of all samples used (lines 177-184; new Supplemental fig S1).**

   line 208; please explain why it is necessary to limit sequence coverage, by sub-sampling reads

**Reply: We have updated the text to explain why sub-sampling is necessary (line 239-240).**

   line 184; The nimblegen oligo library was designed against the reference, what is the sequence identity tolerance of this with respect to successful hybridisation?

**Reply: The probes are proprietary which does not allow us to determine this. However, we were told by the company’s tech support that average sequence divergence relative to the probe of up to 10% was well tolerated (lines 213-217).**

   line 274-9: High sensitivity relates to how strains sequence map to the reference, but what about strain sequence that is missing from the reference? You quantify sequence that assembles de novo without mapping to the reference, but what about sequences that were not captured at all? Are you able to compare assemblies of 454 and illumina reads for the same DNA?

**Reply: We appreciate this insightful suggestion. We have now added text in the results section regarding sensitivity relative to the orthologous ref genome (lines 441-453) and added the corresponding data to Supplemental Table S4 (previously Table S6). Sensitivity of the capture approach relative to the orthologous (454-based) genome was calculated as the percent of the 454-based genome assembly with coverage from capture-based reads.**

   line 700; please comment on what could be done to adapt the oligo array design to ensure tpr and other variable regions are captured from clinical samples.

**Reply: We agree with the editor that this is an important consideration for any future changes to the probe design. The original probe set was designed conservatively, given the unknown impact of repeats on capture efficiency, or possible fragmentation of target DNA fragments. We have now included additional comment in a discussion paragraph on this topic starting in line 865.**

   Table2; I think the table should be ordered somehow to help the reader, perhaps on decreasing variability

**Reply: The table is currently ordered alphabetically by gene identifier, which is based on the location of the gene in the genome. We clarified this in the footnotes of the table.**

   line 578; "Morrison in prep", if reference not in print then please provide a citable DOI or arXiv URL.
   line 608; "Palmateer in prep", if reference not in print then please provide a citable DOI or arXiv URL.

**Reply: We removed both references to findings in unpublished work.**

Reviewer's Responses to Questions
  Key Review Criteria Required for Acceptance?
   As you describe the new analyses required for acceptance, please consider the following:

Methods
   -Are the objectives of the study clearly articulated with a clear testable hypothesis stated?
   -Is the study design appropriate to address the stated objectives?
   -Is the population clearly described and appropriate for the hypothesis being tested?
   -Is the sample size sufficient to ensure adequate power to address the hypothesis being tested?
   -Were correct statistical analysis used to support conclusions?
   -Are there concerns about ethical or regulatory requirements being met?

   Reviewer #1: The objectives and study design was clearly presented. I have no issues.

   Reviewer #2: (No Response)

   Reviewer #3: The methodology appears appropriate - see comments

   --------------------

   Results
   -Does the analysis presented match the analysis plan?
   -Are the results clearly and completely presented?
   -Are the figures (Tables, Images) of sufficient quality for clarity?

   Reviewer #1: Results and figures are clear and well presented. 

   "Raw sequence reads and corresponding genome assemblies available from NCBI under project number PRJNA16138. (In progress)" - please ensure that all assemblies are indeed available.  I could only see the T. Parva Muguga strain genome - maybe I missed it though.

**Reply: Raw sequence reads and corresponding genome assemblies have been submitted and will soon be available from NCBI, under BioProject accession numbers PRJNA656576 (T. parva Muguga_BV115), PRJNA656578 (T. parva Uganda), PRJNA656581 (T. parva Marikebuni) and PRJNA656583 (T. parva lawrencei, aka, Buffalo_3081).** **Accessions will be made publicly available upon manuscript publication.**

   Reviewer #2: (No Response)

   Reviewer #3: see comments

   --------------------

   Conclusions
   -Are the conclusions supported by the data presented?
   -Are the limitations of analysis clearly described?
   -Do the authors discuss how these data can be helpful to advance our understanding of the topic under study?
   -Is public health relevance addressed?

   Reviewer #1: It seems that a disadvantage to the DNA-capture approach is the failure to cover members of multigene families – please discuss this a bit further. The probe set used for DNA capture covers >95% of the genome, and only 53 genes were completely without probe coverage. Of those 53 genes, many belong to multigene families. I would like to see a bit more discussion about the significance of the Tpr (and SVSP and other) multigene families in Theileria biology – in particular what is known about the biological function of Tpr multigene families, and to what extent have Tpr loci been sequenced in other Theileria isolates / species / following different methods.

**Reply: We agree with the reviewer that this is an important limitation of the current probe set. We addressed this comment with additional comment in a discussion paragraph starting on line 865.**

   The authors allude to a the high level of genetic divergence between cattle and buffalo derived T. parva having “clear implications for vaccine development” – I’d appreciate a bit more detail here and a more in-depth discussion of what these implications are.

**Reply: We agree with the reviewer that this statement was vague, and the point is important. We sought to not only demonstrate the capability of our capture approach in the generation of genomic data for these *T. parva* samples, but also convey that we can now better detect the genetic diversity that exists within these strains using this approach. We expanded upon this statement in the abstract (L55-57). We also reorganized considerably the last three paragraphs of the Discussion and expanded on the implications of our results to vaccine development (L981-996).**

   Reviewer #2: (No Response)

   Reviewer #3: see comments

   --------------------

   Editorial and Data Presentation Modifications?
   Use  this section for editorial suggestions as well as relatively minor modifications of existing data that would enhance clarity. If the only modifications needed are minor and/or editorial, you may wish to recommend “Minor Revision” or “Accept”. 

   Reviewer #1: Line 260:  “The capture probe set targets almost completely span the nuclear and apicoplast genomes…» - delete either “targets” or “Span”

**Reply: We deleted the word “targets”.**

   Reviewer #2: (No Response)

   Reviewer #3: see comments

   --------------------

Summary and General Comments
   Use  this section to provide overall comments, discuss strengths/weaknesses of the study, novelty, significance, general execution and scholarship. You may also include additional comments for the author, including concerns about dual publication, research ethics, or publication ethics. If requesting major revision, please articulate the new experiments that are needed.

**Reviewer #1:**

In this manuscript, the authors describe a capture-based target enrichment approach that enabled de novo assembly of almost-complete Theileria parva genomes derived from infected host cell lines. Importantly, the first buffalo-derived T. parva genome assembly is presented and compared with the cattle-derived parasites. A remarkably high level of genetic divergence (as measured by non-synonymous nucleotide diversity per gene) was observed between buffalo and cattle-derived parasites. The capture method uses custom-designed biotinylated oligonucleotides for hybridization to the target sequence – in this case the probes were designed on the T. parva Muguga reference genome. The sensitivity and specificity of this capture method was very impressive. Sequencing was performed using Illumina technology.  

   In my opinion the analysis of the new genome assemblies was robust, and the data well laid out and clearly presented. In particular the authors present the first annotated genome assembly from buffalo-derived T. parva – this will be a hugely valuable resource, in particular in the context of vaccine design. I only have a few very minor points.

**Reply: We thank reviewer 1 for the thoughtful comments on the quality of this capture method and the supporting analysis. We have addressed this reviewer’s questions above.**

**Reviewer #2:**

In their study “Capture-based enrichment of Theileria parva DNA enables full genome assembly of first buffalo-derived strain and reveals exceptional intra-specific genetic diversity” the authors describe a DNA capture technique that lead to the assembly and analysis of the buffalo-derived T. parva genome. I have no major issues, but I do have a number of more minor comments that should be considered by authors and editor before final acceptance of the manuscript.

**Reply: We thank reviewer 2 for the thoughtful comments and have addressed their comments and concerns below.**

   P2L49: How much larger is the genome? Has it also more predicted genes? This information could be added to the abstract.

**Reply: We have now added this information to the abstract.**

   P2L52: FST – abbreviation should be explained.

**Reply: Done. Also expanded explanation of Fst in Methods (lines 284-287).**

   P2L55: What is the advantage and what are the previous methods used (see also comment below)?

**Reply: We expanded briefly on the advantages in the sentence reviewer 2 refers to at the end of the abstract and have expanded on our discussion of the advantages over previous methods in greater length, starting in line 814 (see comment below).**

   P2L59/60 and P3L86/87: I doubt that this number is correct. It appears very high. Please give a timely reference (e.g. in the introduction).

**Reply: To our knowledge, there have been no recent surveys of the mortality levels of cattle across the whole endemic region, caused by *T. parva*. The most recent estimate of the impact of the disease that is available was derived in the early 1990s, as discussed in citation #13 (Norval *et al.* 1991). We have removed this value from the authors’ summary, clarified its context in the introduction and also now include a current estimate of animals are risk and total losses, available from GALVmed, in the author summary (line 66) and the introduction (lines 96-98)**

   P5L115-125: The procedure using aerolysin (cp. Baumgartner et al. Microbes and Infection 1999) appears to be highly efficient and produces good quality parasite DNA for NGS approaches. This alternative approach should be more critically discussed and compared by the authors. In particular as it was successfully used for NGS approaches before (cp. Hayashida et al. 2013).

**Reply: We agree with the reviewer that it is important to emphasize what makes our work unique from previous studies. We discuss some of the drawbacks of other methods that initially motivated us to develop and implement the capture approach in the lines the reviewer points to. We have added to the relevant section of the discussion to expand on the advantages, based on our results (L814).**

   P6L164: Why was the buffalo and Muguga strain not cloned prior to analysis? Most field isolates are multiclonal. Given that high SNP ratios can be observed in particular in highly variable genes such as SVSPs between strains, are multiclonal isolates not complicating SNP analyses?

**Reply: Initially, we were referring to the history of the isolates, as per the original reference describing their generation. We now include an expanded history of the cell lines used. In fact, BV115 is a cloned stabilate of the Muguga stock, and we now mention that (lines 178-181). Regarding potential polyclonality and its potential impact on analyses, the SNP calling pipeline most frequently implemented in these types of studies (including here) calls the major allele at each position, to address the potential confounders of polyclonality, sequencing error, etc. Also, the likelihood that reads containing any minor allele impact an assembly is relatively minor because the signal comes primarily from the most represented allele, especially once the assemblies are polished with Pilon (as they were here), which takes as input the info from all reads.**

    P6L165/166: Since Hayashida et al. (2013 DNA Research) have already sequenced and analysed the same buffalo-derived strain, it appears important to further stress the advantage of the current study (such as the genome assembly).

**Reply: While we had assumed that the buffalo isolate we sequenced was the same that was analyzed before by Hayashida, when we mapped the reads from the Buffalo-LAWR from that study to our assembly we realized that the they are actually quite distinct. Accordingly, we changed the designation of our sample throughout and use the stabilate number, and now added a new paragraph in the results section to reflect this, starting in line 579. We also stress further the advantage of the method followed here over that of Hayashida et al. (2013) in the discussion, starting on line 814. We also originally noted on line 610 the added power of detecting SNPs using assembly comparison over genotyping by read mapping, as done by Hayashida et al. (2013).**

   P14, Figure 2: Legends and annotations are in part too small.
**Reply: We have increased the size of the text in Figure 2.**

**Reviewer #3**

The authors report the first use of capture-based target enrichment to sequence and assemble the genomes of Thieleria parva parasites from infected mammalian cells. Although this approach has been previously applied to sequencing the Plasmodium genome (e.g. Bright et al. 2012), several features of the experimental design for Theileria have resulted in impressive levels of specificity and sensitivity and coverage.

The sequencing data allowed de novo genome assembly providing an important proof-of-concept and a resource for future studies of parasite genotypes and population genetics. The generation of the first buffalo-derived T.parva genome is interesting because of the distinct infection symptoms and could be relevant to vaccine development. The sequencing data reveal differences in the genome size and genetic diversity between cattle- and buffalo-derived parasites.

Minor points

- It is a shame that the authors only tested their approach on established infected cell lines. They should at least comment on the feasibility of testing on clinical samples from infected cows and ticks with respect to sensitivity and the limited sample material. They should also comment on the cost, time-requirement and scalability of this technology.

**Reply: This is now addressed in the first paragraph in the discussion (L814-L839).**

- Figure 2 is so small that the images will be hardly readable in the final print form.

**Reply: We have increased the size of the text in Figure 2.**

- Page 19: the authors report 24 new genes found in the Buffalo assembly. Are the 19 genes, representing copies of T. parva genes, found in particular locations adjacent to their orthologues? Are the 5 genes with T. annulata and T.orientalis homologues founds in regions syntenic with the other species? All these 24 genes should be listed in a table with T.annulata and T.orientalis reference names.

**Reply: We have updated Supplemental Table S9, which previously listed the potential new genes identified in *T. annulata* and *T. orientalis*, to meet the request of the reviewer. We now list all newly identified potential genes in the buffalo assembly, and includes the NCBI accession number as well. We also note whether or not they are in close proximity to other current copies or syntenic to genes from other species, in the paragraph discussing these results (L5561).**

- Page 28: The manuscript should have consistent citation (by numbers) e.g. lines 685, 686, 687 etc.

**Reply: We have updated the citations to be consistent with the rest of the document.**

The authors should comment on the occurrence and predicted effects of specific SNPs in parasite genes encoding proteins shown to be important for host-parasite interactions and drug targeting: e.g. p67, Tp1, Tp2, UB05, Pin1, cytochrome B, p104, Ta9, MISHIP, TashAT2 etc.

**Reply: We have added comment on this subject in the results section pertaining to the level of polymorphism in antigens based on the three non-reference samples analyzed here (L700-704). More extensive characterization of polymorphism is already available in papers focused on those specific genes (eg, PMIDs 25673078, 29729680, 21559495, 21621920), which is more informative than can be done here. We are now conducting an expanded study based on the method reported in the current paper, which will involve a few dozen cattle- and buffalo-derived samples. This will allow us to investigate fixed differences between the two parasite populations in all these genes, which will be very informative.**
